# New Artificial Intelligence Score and Immune Infiltrates as Prognostic Factors in Colorectal Cancer With Brain Metastases

**DOI:** 10.3389/fimmu.2021.750407

**Published:** 2021-10-18

**Authors:** Violaine Randrian, Amandine Desette, Sheik Emambux, Valentin Derangere, Pauline Roussille, Eric Frouin, Julie Godet, Lucie Karayan-Tapon, François Ghiringhelli, David Tougeron

**Affiliations:** ^1^ Hepato-Gastroenterology Department, CHU Poitiers, Poitiers, France; ^2^ Université de Poitiers, CHU Poitiers, INSERM, PRODICET, Poitiers, France; ^3^ Université de Poitiers, CHU Poitiers, INSERM, LNEC, Poitiers, France; ^4^ Medical Oncology Department, CHU Poitiers, Poitiers, France; ^5^ Plateforme de Transfert en Biologie Cancérologique, Département de Biologie et de Pathologie des Tumeurs, Centre de Lutte Contre le Cancer Georges-François Leclerc, Dijon, France; ^6^ Radiotherapy Department, CHU Poitiers, Poitiers, France; ^7^ Pathology Department, CHU Poitiers, Poitiers, France; ^8^ Université de Poitiers, CHU Poitiers, LITEC, Poitiers, France; ^9^ Cancer Biology Department, CHU Poitiers, Poitiers, France; ^10^ INSERM U1231, Dijon, France

**Keywords:** colorectal cancer, brain metastases, anti-tumoral immunity, tumor infiltrated lymphocytes (TILs), prognostic factors, CD3

## Abstract

Incidence of brain metastases has increased in patients with colorectal cancer (CRC) as their survival has improved. CD3 T-cells and, lately, DGMate (DiGital tuMor pArameTErs) score, have been identified as prognostic factors in locally advanced CRC. Until now, there is no data concerning the prognostic value of these markers in patients with CRC-derived brain metastases. All consecutive patients with CRC-derived brain metastases diagnosed between 2000 and 2017 were retrospectively included. Staining for CD3, CD8, PD-1, PD-L1 and DGMate analyses were performed using tissue micro-array from primary tumors and, if available, brain metastases. All in all, 83 patients were included with 80 primary tumor samples and 37 brain metastases samples available. CD3 and CD8 T-cell infiltration was higher in primary tumors compared to brain metastases. We observed a significant higher DGMate score in rectal tumors compared to colon tumors (p=0.03). We also noted a trend of higher CD3 T-cell infiltration in primary tumors when brain metastases were both supra and subtentorial compared to brain metastases that were only subtentorial or supratentorial (p=0.36 and p=0.03, respectively). No correlation was found between CD3 or CD8 infiltration or DGMate score in primary tumors or brain metastases and overall survival (OS) in the overall population. In patients with rectal tumors, a high DGMate score in brain metastases was associated with longer OS (13.4 ± 6.1 months versus 6.1 ± 1.4 months, p=0.02). High CD3 T-cell infiltration in brain metastases was associated with lower OS in patients with supratentorial brain metastases (9.8 ± 3.3 months versus 16.7 ± 5.9 months, p=0.03). PD-L1 overexpression was rare, both in primary tumors and brain metastases, but PD-L1 positive primary tumors were associated with worse OS (p=0.01). In contrast to breast and lung cancer derived brain metastases, CD3 and CD8 infiltration and DGMate score are not major prognostic factors in patients with CRC-derived brain metastases.

## Introduction

The prognosis of patients with metastatic colorectal cancer (mCRC) has improved and median overall survival (OS) is now about three years. In parallel to this OS improvement, the incidence of unusual metastatic sites such as brain metastases (BM) has increased (1). Mostly metachronous, BMs derived from CRC are diagnosed about two years after the primary tumor diagnosis and are usually associated with a *RAS* mutation (2, 3). CRC-derived BMs remain associated with a poor prognosis with 5 months of median OS (4).

Immune infiltration is a known prognostic factor in locally advanced CRC and will perhaps be used in the near future as a prognostic factor to determine modalities of adjuvant chemotherapy (5, 6). The percentage of CD3+ T cells at the invasive margin of locally advanced CRC is also a predictive factor of metachronous metastases (7). It also remains a robust prognostic factor at the metastatic stage (8). Furthermore, CD3+ T lymphocytes are the main type of tumor-infiltrating lymphocytes (TIL) identified in BMs of various primary tumors. This infiltration correlates with prolonged OS in BMs derived from lung cancer, breast cancer, melanoma or renal cell cancer (9). In CRC-derived BMs, data are lacking since BMs from CRC are rare (1 to 5% of CRC) (3). This is of great interest as prognosis in mCRC is correlated with infiltration of the least-immune infiltrated metastases and BMs are supposed to be poorly infiltrated by immune cells (10). Therefore, more biological insight is needed to characterize dynamic and prognostic significance of immune infiltration, especially by CD3+ T cells, in this rare subgroup of CRC with BMs.

An artificial intelligence software device, using a LASSO algorithm called DiGital tuMour pArameTErs (DGMate), was shown in the PETACC08 study to predict the prognosis of locally advanced colon cancers (stage III) (11). DGMate score is a set of texture parameters extracted from the CRC tissue. When combined with CD3 staining, it overwhelms immune score performance in predicting the outcome of locally advanced colon cancer. Indeed, a predictive nomogram based on DGMate, CD3 TIL and clinical variables has identified a group of patients with less than 10% relapse risk and another group with a 50% relapse risk in stage III CRC. These tools are not yet validated in mCRC. We analyzed both CD3 infiltration and DGMate score in a rare series of CRC-derived BM to assess whether CD3 infiltration and/or DGMate score were prognostic factors in CRC-derived BMs.

## Materials and Methods

### Patients

This study was conducted on samples available from patients included in the study previously published by Roussille P et al. (2, 3). All consecutive patients with BM from CRC, diagnosed from 2001 to 2016, were identified in our institution using our clinical report database. Inclusion criteria were age over 18 years, histologically confirmed CRC and histologically or radiologically confirmed BM by computed tomography scan (CT-scan) and/or magnetic resonance imaging (MRI) were included. Our institution’s Ethics Committee approved the study (DC-2008-565). The study was performed according to the principles of the Declaration of Helsinki.

Microsatellite stable/instable status (MSS/MSI), *KRAS*, *NRAS* and *BRAF V600E* mutational statuses were determined as previously described (2).

### Tissue Microarray Construction and Immunohistochemistry

Formalin-fixed paraffin-embedded (FFPE) blocks were used for tissue microarray (TMA) construction using four biopsy cores of 1 mm diameter per tumor in the tumor center (MTA Booster^©^ version 1.01, Alphelys, Paris, France). Both primary tumors (PT) and BMs, if available, were included in the TMA.

**Figure 1:**
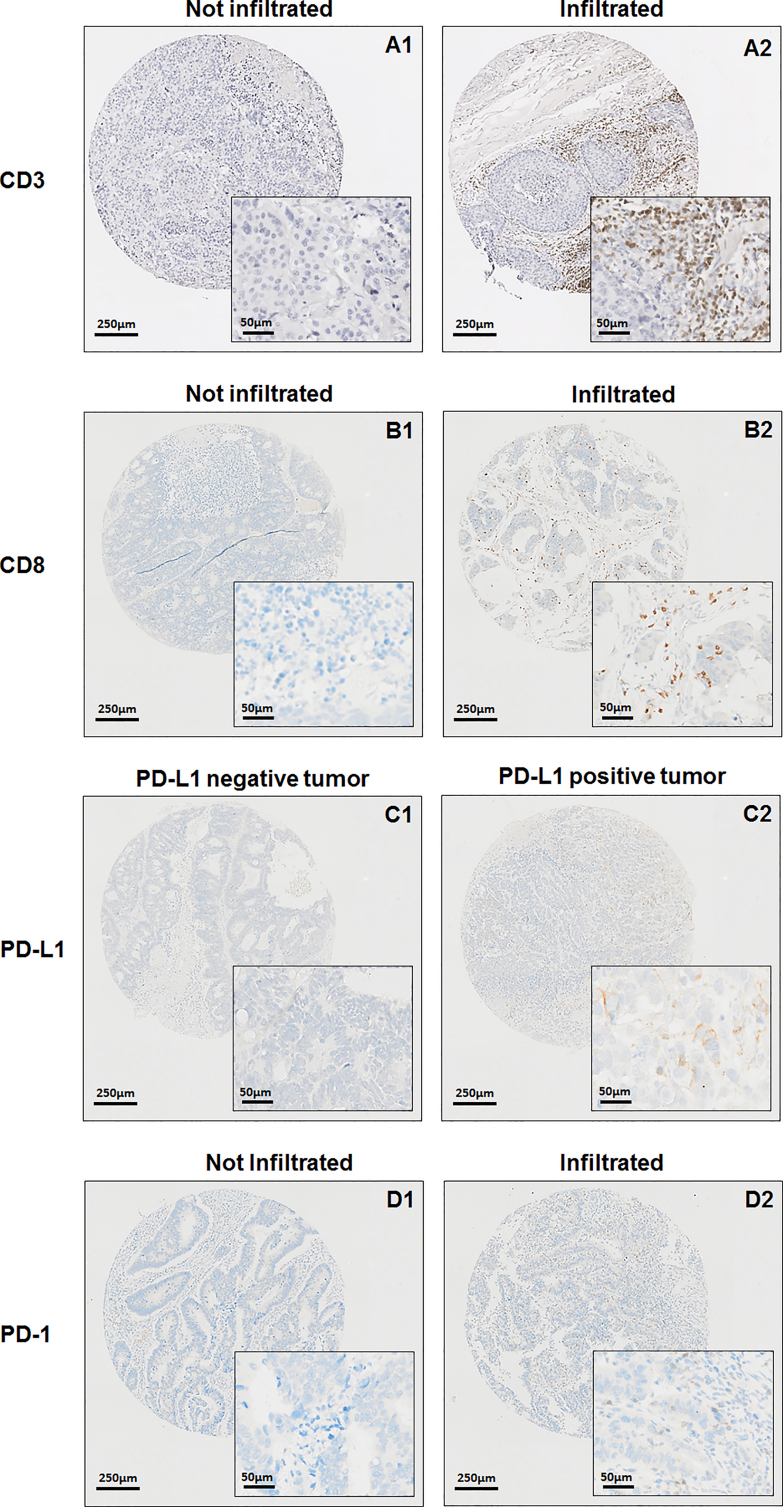
Median overall survival after brain metastases diagnosis. Kaplan-Meier method was used to determine OS.

IHC was carried out on paraffin-embedded 3-µm thick TMA sections with antibodies directed against Programmed death-1 (PD-1) (clone NAT105, Roche^©^), Programmed death-ligand 1 (PD-L1) (clone SP263, Roche^©^), CD3 (clone F7.2.38, Agilent^©^) and CD8 (clone C8144B, Dako^©^) according to the manufacturer’s instructions. Once counterstained and permanently mounted, slides were scanned with a Nanozoomer HT2.0 (Hamamatsu Photonics) at ×20 magnification to generate a whole slide imaging (WSI) file in ndpi format (**Figure 1**).

For each TMA core, CD3 positive cells were detected using QuPath^©^ software (12) and exported as a number of positive cells by mm² (TMA core area = 1,13 mm²). DGMate score was calculated for each tumor core as described by Reichling and colleagues (11). Briefly, using QuPath^©^ software, the whole slide was tiled using a DoG superpixel strategy. For each tile QuPath^©^ is able to measure and export 127 parameters related to color, texture or pixel environment within the tile. These parameters are used in a random forest prediction model, called Coloclass (11), to classify tiles in several tissue classes such as tumor, immune patch, healthy and stroma for instance. Next, restricting information to tiles classified as tumors, a score predictive of relapse-free survival in stage III colon cancer, coined DGMate, was estimated through a Cox regression model with lasso method to select a minimum set of predictive parameters. The CD3 lymphocyte surface area and DGMate were quantified automatically both in the tumor core of PT and BM.

PD-1 IHC was considered positive when ≥1% of intra-epithelial tumor infiltrating lymphocytes (TILs) were stained. PD-L1 immunostaining was considered positive when ≥1% of tumor cells had membranous staining. CD3 and CD8 staining were also analyzed as the percentage of both intra-tumoral and stromal CD3 and CD8 positive lymphocytes over the total immune cells (13).

### Statistical Analysis

Continuous variables were described with median, standard deviation (SD) and range. Qualitative variables were described with frequency and percentage. Comparisons of characteristics were performed with the non-parametric Mann-Whitney (2 groups) or Kruskall-Wallis (3 groups or more) tests for continuous variables and the chi-square test or Fisher’s exact test for qualitative variables. Correlation was determined calculating Spearman’s rank-order coefficient.

The primary endpoint was OS, defined by the time between BM diagnosis and death, whatever the causes. Survival curves and 95% confidence intervals (CI) were determined using the Kaplan-Meier method. Predictive factors of OS were evaluated using the log-rank test for univariate analysis and variables with p values ≤0.10 in univariable analyses were included in multivariate analysis using a Cox regression model.

The level of significance was set at a p value of 0.05. All statistical tests were two-sided. Statistical analyses were performed using Statview^©^ 4.0 software (SAS Institute Inc., Cary, NC, USA).

## Results

### Population

Eighty-three patients were included with PT samples available in 80 cases and BMs tissues available for 37 patients. Samples from both PT and BMs were available for staining analysis for 34 patients. Median age at BM diagnosis was 66.8 years old (**Table 1**). Most patients had a T3 (64.0%) or a T4 (25.3%) tumor with lymph nodes invasion (72.4%). At CRC diagnosis most tumors were stage III or IV (79.2%). *RAS* was mutated in 61.5% of cases and *BRAF* was mutated in 6.4% of cases.

**Table 1:** Patients, primary tumors and brain metastases characteristics.

n = 83	n (%)
**Age at BM diagnosis (median, min-max)**	66.8 (36.8-87.1)
**Gender**	
Male	53 (63.9%)
Female	30 (36.1%)
**Site of primary tumor**	
Ascending colon	19 (22.9%)
Descending colon	26 (31.3%)
Rectum	34 (41.0%)
Bifocal tumor	4 (4.8%)
**Tumor grade**	
Well differentiated	24 (33.8%)
Moderately differentiated	38 (53.5%)
Poorly differentiated	9 (12.7%)
Missing data	12
**Stage at initial CRC diagnostic**	
I	4 (4.9%)
II	13 (15.9%)
III	28 (34.1%)
IV	37 (45.1%)
Missing data	1
**Primary tumor resection**	73 (88.0%)
**ECOG performance status at BM diagnosis**	
0	14 (17.5%)
1	28 (35.0%)
2	21 (26.3%)
3	17 (21.2%)
4	0
Missing data	3
**Lung metastases at BM diagnosis**	
Yes	59 (71.1%)
No	24 (28.9%)
**Liver metastases at BM diagnosis**	
Yes	36 (43.4%)
No	47 (56.6%)
**Site of BM**	
Supratentorial only	48 (57.8%)
Subtentorial only	17 (20.5%)
Supra and subtentorial	18 (21.7%)
**Number of brain metastases**	
Single	42 (50.6%)
Multiple	41 (49.4%)
**Interval between primary tumor and BM diagnosis**	
Synchronous	6 (7.2%)
Metachronous	77 (92.8%)
**Interval between BM and extracranial disease (n=75)**	
Synchronous	15 (20.0%)
Metachronous	60 (80.0%)
**Molecular characteristics**	
* RAS*: Wild-type/Mutated/Missing data	30 (38.5%)/48 (61.5%)/5
* BRAF*: Wild-type/Mutated/Missing data	73 (93.6%)/5 (6.4%)/5
MMR status: MSI/MSS/Missing data	4 (5.5%)/69 (94.5%)/10

CRC, colorectal cancer; BM, brain metastases; PT, primary tumor; SD, standard deviation; MMR, mismatch repair; MSS, microsatellite stable; MSI, microsatellite instability; ECOG, Eastern Cooperative Oncology Group.

Among the 83 patients included, 96.4% had neurologic symptoms that led to the BM diagnosis. At BM diagnosis most patients had extracranial metastases (ECM) (90.4%) and 81.9% had already received at least one line of chemotherapy for their metastatic disease. Most patients had metachronous BM from PT diagnosis (92.8%) or from ECM diagnosis (80.0%). A minority of patients had synchronous BM at diagnosis of metastatic disease (20.0%). Median interval between BM diagnosis and PT diagnosis was 35.1 ± 3.4 months. Median interval between BM diagnosis and ECM diagnosis was 21.2 ± 2.7 months.

About half of patients had single BM (50.6%), mostly supratentorial only (57.8%). At BM diagnosis half patients had an Eastern Cooperative Oncology Group performance status (ECOG PS) at 2 or more (47.5%).

All in all, 37 patients (44.6%) underwent BM surgery. Decision of BM surgery was decided during a multidisciplinary team meeting based on the performance status of the patient, the expected OS, the number, size and location of BM. Most patients (83.0%) underwent radiotherapy of the BM. Among the patients treated with radiotherapy, 23.5% were treated by stereotactic radiosurgery and 69.1% were treated with whole brain radiotherapy. The remaining patients received local radiotherapy without using stereotactic radiosurgery. Among the patients with BM surgery, most have undergone previous chemotherapy for the metastatic disease (59.5%) and adjuvant radiotherapy after BM surgery (94.6%).

### CD3 T-Cell Infiltration and DGMate Score in Primary Tumor and Brain Metastases

**Figure 2:**
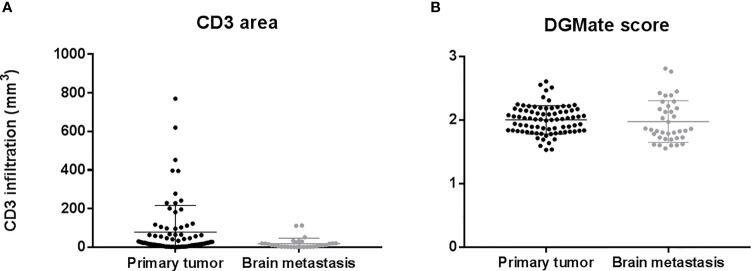
CD3 infiltration **(A)** and DGMate **(B)** in primary tumor and brain metastases.

CD3 T-cell infiltration was higher in PT as compared to BM (78.9/mm^3^ versus 19.1/mm^3^, p=0.0071) (**Figure 2**). We observed no correlation of CD3 T-cell infiltration between BM and PT (Rho=0.29, p=0.13).

Concerning the rate of the DGMate score there was no statistical difference between PT and BM (p=0.86). We observed a strong correlation between the DGMate score in PT and the DGMate score BM (Rho=0.62, p=0.0004).

### Correlation Between CD3 T-Cell Infiltration and DGMate Score With Patient and Tumor Characteristics

Age, gender, tumor grade, stage at initial CRC diagnostic, T stage, N stage, interval between metastases and PT diagnosis, interval between BM and PT diagnosis, interval between BM and ECM diagnosis, *RAS* status, *BRAF* status and MMR status were not associated with CD3 T-cell infiltration in PTs (**Table 2**). CD3 T-cell infiltration in PTs increased with T stage from 24.6/mm^3^ for T1 to 100.7/mm^3^ for T4 but was not significant (p=0.43). Concerning DGMate score in PTs, using the same patient and tumor characteristics, no statistically difference was observed. DGMate score in PTs increased with T stage from 1.85 for T1 to 1.98 for T4 but was not significant (p=0.31). There was no significant difference in CD3 infiltration or DGMate score in PTs according to the metachronous or synchronous status of the BM from the diagnosis of PT or ECM.

**Table 2:** Correlation between CD3 T-cell infiltration and DGMate score with patient and tumor characteristics.

	CD3 area		DGMate score	
	Primary tumor (p value)	Brain metastasis (p value)	Primary tumor (p value)	Brain metastasis (p value)
**Age (continuous variable)**	0.81	0.09	0.84	0.41
**Gender (male vs female)**	0.37	0.58	0.22	0.92
**Site of primary tumor (ascending colon vs descending colon vs rectum)**	0.53	0.42	0.11	0.54
**Tumor grade (well and moderately differentiated vs poorly differentiated)**	0.08	0.10	0.21	0.26
**Stage at initial CRC diagnostic (I vs II vs III vs IV)**	0.74	0.94	0.99	0.43
**T stage (T1 and T2 vs T3 vs T4)**	0.43	0.31	0.44	0.65
**N stage (N0 vs N1 vs N2)**	0.39	0.22	0.24	0.55
**Interval between metastases diagnosis and PT (continuous variable)**	0.29	0.73	0.59	0.16
**Interval between BM diagnosis and PT (continuous variable)**	0.17	0.36	0.27	0.16
**Interval between BM and ECM (continuous variable)**	0.27	0.16	0.19	0.28
***RAS* status (mutated vs wild-type)**	0.68	0.43	0.70	0.30
***BRAF* status (mutated vs wild-type)**	0.37	0.58	0.79	0.11
**MMR status (MSS vs MSI)**	0.68	0.35	0.80	0.08

vs, versus; ECM, extracranial metastases; PT, primary tumor; BM, brain metastases; MMR, mismatch repair; MSI, microsatellite instability; MSS, microsatellite stability.

CD3 T-cell infiltration or DGMate score in BMs were not statistically different according to patient or tumor characteristics (**Table 2**).

We observed non-significantly higher CD3 T-cell infiltration in rectal tumors compared to colon tumors (108.7 ± 30.4/mm^3^ versus 54.9 ± 15.9/mm^3^, p=0.26) and a significantly higher DGMate score (2.1 ± 0.1 versus 1.9 ± 0.1, p=0.03) (**Table 3**). When BMs were both supra and subtentorial, mean CD3 T-cell infiltration in PTs (137.1 ± 42.8/mm^3^) was higher than when BMs were subtentorial only (85.7 ± 31.2/mm^3^) or supratentorial only (53.9 ± 17.5/mm^3^) (p=0.36 and p=0.03, respectively). The same trend was observed with CD3 T-cell infiltration in BMs. There was a non-significant increase of CD3 T-cell infiltration in PT and BM when BMs were multiple (p=0.40 and p=0.23, respectively) (**Table 3**). We also observed a trend of higher DGMate score in BM in patients with multiple BMs (2.2 ± 0.1 versus 1.9 ± 0.1, p=0.07).

**Table 3:** CD3 T-cell infiltration and DGMate score in primary tumor and brain metastases according to BM site and BM numbers.

	Primary Tumor			Brain Metastases		
	n	CD3 area*	DGMate	n	CD3 area*	DGMate
**Site of primary tumor**						
**Colon**	42	54.9 ± 15.9	1.9 ± 0.1	24	16.0 ± 17.5	2.0 ± 0.1
**Rectum**	34	108.7 ± 30.4	2.1 ± 0.1	12	28.2 ± 12.5	2.0 ± 0.1
**Site of BM**						
**Supratentorial only**	46	53.9 ± 17.5	2.0 ± 0.1	23	15.3 ± 5.5	1.9 ± 0.1
**Subtentorial only**	16	85.7 ± 31.2	2.0 ± 0.1	13	17.4 ± 5.1	2.0 ± 0.1
**Supra and subtentorial**	18	137.1 ± 42.8	2.0 ± 0.1	1	113.2	2.2
**Number of BM**						
**Unique BM**	39	64.6 ± 21.3	2.0 ± 0.1	30	15.8 ± 4.7	1.9 ± 0.1
**Multiple BM**	41	92.1 ± 22.6	2.0 ± 0.1	7	32.9 ± 16.6	2.2 ± 0.1

* Mean CD3 infiltration was expressed by mm^3^.

### CD8 T-Cell Infiltration and Expression of PD-L1 In Primary Tumor and Brain Metastases

PTs had CD8 positive T-cells in most cases (93.4%) with a mean of 13.7% CD8+ lymphocyte infiltrates (median 10.0%, range 0-70.0%). BM CD8 positive T-cells were less frequent (62.9%, n=22/35), with a mean of 8.6% of CD8+ lymphocyte infiltrates (median 3.0%, range 0-50.0%). While there was a correlation between CD8 T-cell infiltration in BM and PT (Rho=0.37, p=0.01), CD8 T-cell infiltration was higher in PT as compared to BM (p=0.03).

Primary tumors with PD-1 positive TILs were 13.3% but no BM with PD-1 positive TILs was found in the available samples. We observed only 6.8% PTs with PD-L1 positive TILs and there were two BMs with PD-L1 positive TILs (n=2/35, 5,7%). Both BMs with PD-L1 positive TILs had PTs with no PD-L1 positive TILs. Among the PTs with PD-L1 positive TILs, only one had an available BM sample and it was negative for PD-L1 TILs.

### Prognostic Value of Immune T-Cell Infiltration and DGMate Score

**Figure 3:**
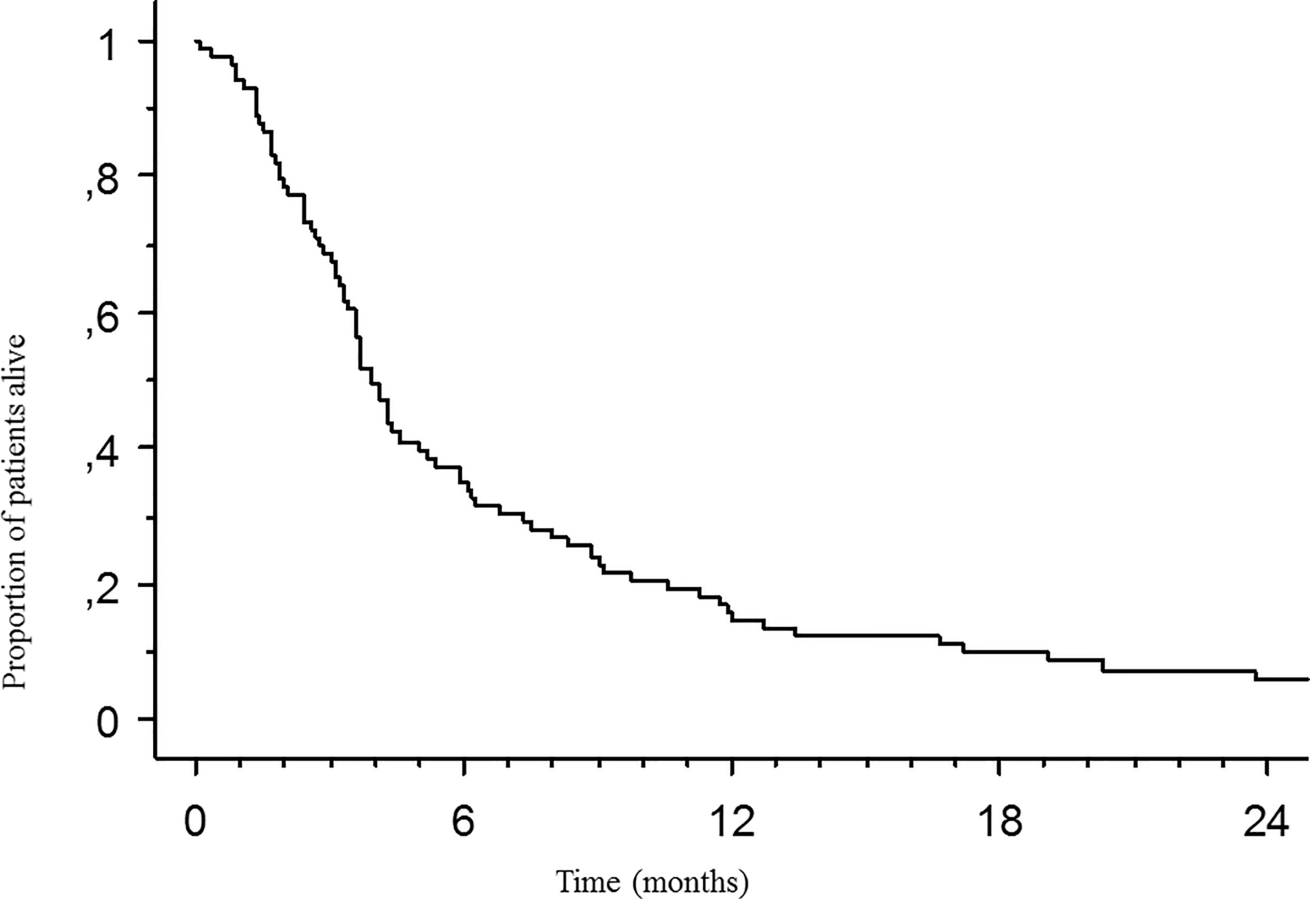
Median overall survival (OS) after brain metastasis diagnosis. Kaplan Meier method was used to determine OS.

Median OS after PT diagnosis was 41.0 ± 1.5 months. Median OS after BM diagnosis was 3.9 ± 0.5 months (**Figure 3**). Patient and tumor characteristics associated with OS after BM diagnosis were age, site of primary tumor, ECOG PS, *BRAF* status, site of BMs, number of BMs and lung metastases in univariate analysis (**Table 4**). Neither CD3 infiltration nor DGMate score in PT or BM was correlated with OS. In multivariate analysis, only ECOG PS 0-1 and absence of lung metastasis were associated with better OS.

**Table 4:** Prognostic factors of overall survival from brain metastases diagnosis.

Variables	n	Univariate analysis		Multivariate analysis		
		Median (months)	p value	HR	95% CI	p value
**Gender**			0.98*			0.67
Male	53	3.9		1		
Female	30	4.1		0.9	0.5-1.5	
**Age at BM diagnosis^#^**	83		0.03*	1.0	1.0-1.0	0.82
**Site of primary tumor (n=79)**			0.05*			0.20
Colon	45	4.6		1		
Rectum	34	2.8		1.4	0.8-2.4	
**Tumor grade (n=71)**			0.59			
Well or moderately differentiated	62	3.7				
Poorly differentiated	9	4.6				
**Primary tumor resection**			0.74			
Yes	73	3.7				
No	10	3.9				
**ECOG PS at BM diagnosis**			<0.01*			0.03
0 or 1	42	6.8		1		
2 or 3	38	3.1		2.1	1.1-4.0	
***RAS* status**			0.66			
Wild-type	30	3.6				
Mutant	48	4.1				
***BRAF* status**			0.04*			0.38
Wild-type	73	4.1		1		
Mutant	5	3.3		1.7	0.5-5.6	
**Site of BM**			0.01*			0.27
Supratentorial only	48	4.3		1		
Subtentorial only	17	4.6		0.6	0.3-1.3	
Supra and subtentorial	18	2.8		1.2	0.5-2.9	
**Number of brain metastases**			<0.01*			0.13
Single	42	6.2		1		
Multiple	41	3.1		1.7	0.9-3.2	
**Interval between PT and BM diagnosis**			0.20			
Synchronous	6	11.7				
Metachronous	77	3.9				
**Interval between BM and extracranial disease (n=75)**			0.11			
Synchronous	15	6.3				
Metachronous	60	3.7				
**Lung metastases at BM diagnosis**			<0.01*			0.02
No	24	8.9		1		
Yes	59	3.7		2.0	1.1-3.8	
**Liver metastases at BM diagnosis**						
No	47	4.1	0.17			
Yes	36	3.6				
**CD3 T-cell infiltration in PT^#^**	79		0.15			
**CD3 T-cell infiltration in BM^#^**	31		0.91			
**DGMate score in PT^#^**	80		0.71			
**DGMate score in BM^#^**	37		0.84			
**CD3 T-cell infiltration in PT^¤^**			0.45			
Low	40	3.7				
High	39	3.9				
**CD3 T-cell infiltration in BM^¤^**			0.23			
Low	14	10.6				
High	15	7.5				
**DGMate score in PT^¤^**			0.78			
Low	40	4.1				
High	40	3.3				
**DGMate score in BM^¤^**			0.92			
Low	19	8.0				
High	18	9.8				

HR, hazard ratio; BM, brain metastasis(es); PT, primary tumor; 95% CI, 95% confidence interval; ECOG PS, Eastern Cooperative Oncology Group score performances status.

*variables included in multivariate analysis.

^#^analyses as continuous variable.

^¤^scores split at median.

**Figure 4:**
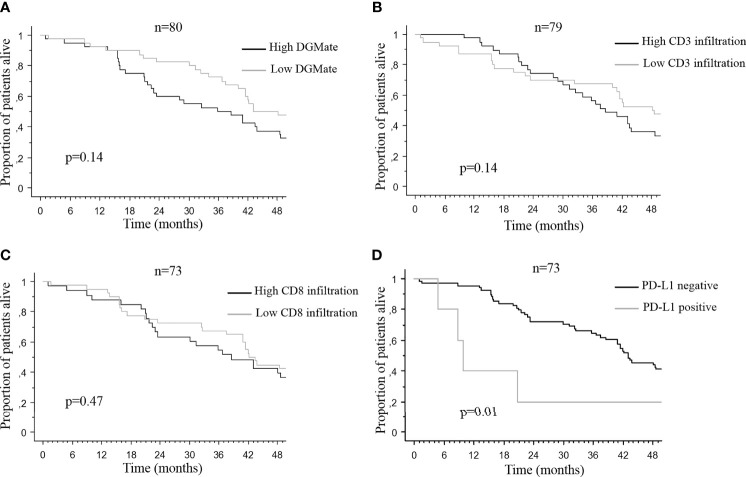
Median overall survival after brain metastases diagnosis according to DGMate score **(A)**, CD3 infiltrating T-cells **(B)**, CD8 infiltrating T-cells **(C)**, and PDL1 positive tumors **(D)** in primary tumor. Kaplan-Meier method was used to determine OS. DGMate, CD3 and CD8 scores were split at median and p valuecalculated using the Logrank test.

Neither CD3 infiltration nor DGMate score in PT or BM was correlated with OS from CRC diagnosis (**Figures 4A, B**). In addition, CD3 infiltration or DGMate score in patients with metastatic or non-metastatic disease at CRC diagnosis had no prognostic impact.

**Figure 5:**
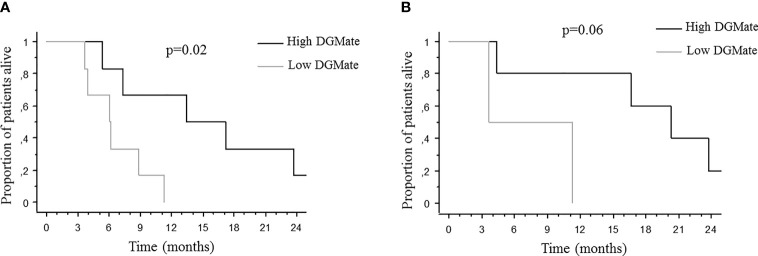
Median overall survival after brain metastases diagnosis according to DGMate score in BM among patients with rectal tumor **(A)** and patients with multiple BMs **(B)**. Kaplan-Meier method was used to determine OS. DGMate score was split at median and p value calculated using the Logrank test.

**Figure 6:**
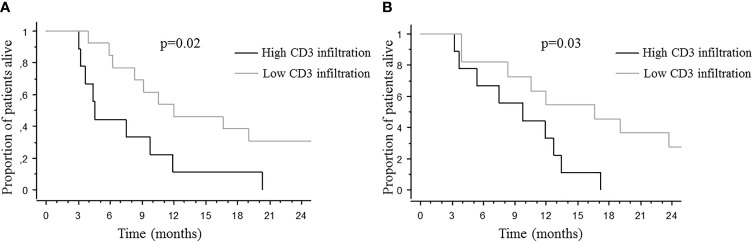
Median overall survival after brain metastases diagnosis according to CD3 T-cell infiltration in BM among patients with colon cancer **(A)** and supratentorial BMs **(B)**. Kaplan-Meier method was used to determine OS. CD3 T-cell infiltration score was split at median and p value calculated using the Logrank test.

Since CD3 T-cell infiltration and/or DGMate scores were different according to PT site, BMs location and BMs number, we looked for a potential prognostic impact in these subgroups. When DGMate score was divided in two groups according to the median, high DGMate score in BM was associated with longer OS in two subgroups: patients with multiple BMs (20.3 ± 3.8 months versus 3.7 ± 4.0 months, p=0.06) and patients with rectal tumor (13.4 ± 6.1 months versus 6.1 ± 1.4 months, p=0.02) (**Figures 5A, B**). When CD3 T-cell infiltration was divided in two groups according to the median, high CD3 T-cell infiltration in BM was associated with lower OS in two subgroups: patients with colon tumor (4.6 ± 2.3 months versus 12.0 ± 5.5 months, p=0.02) and patients with supratentorial BMs (9.8 ± 3.3 months versus 16.7 ± 5.9 months, p=0.03) (**Figures 6A, B**).

PD-L1 positive PTs were associated with worse OS from CRC diagnosis (10.1 ± 6.6 months versus 43.1 ± 1.6 months, p=0.01) (**Figure 4D**). CD8 T-cell infiltration in PT was not correlated with OS (**Figure 4C**). Nor PD-L1 positive BM or CD8 T-cell infiltration in BM was correlated with OS after BM diagnosis.

## Discussion

This large series of 83 CRC patients with BM displayed lower CD3 and CD8 T-cell infiltration in BMs compared with PTs. In PTs there was a trend of higher CD3 T-cell infiltration in rectal tumor, when BMs were both supra and subtentorial and when BMs were multiple. No correlation was found between CD3 or CD8 infiltration in PT or BM and OS in overall population. Patients with high CD3 T-cell infiltration in BMs had a lower OS in two subgroups: patients with colon tumor and patients with supratentorial BMs. In contrast to locally advanced CRC, CD3 and CD8 infiltration and DGMate score were not robust prognostic factor in CRC patients with BM. PD-L1 positive PTs or BMs were rare but PD-L1 positive PTs were associated with worse OS from CRC diagnosis.

Our series of 83 CRC patients with BM had similar patients and tumor characteristics as previously described, i.e. frequent rectal tumor, lung metastases, synchronous metastatic disease and *RAS*-mutated tumors (1, 3). In addition, CRCs are associated with different clinicopathological features according to the type of *RAS* mutation (14). Most CRC-derived BMs were metachronous (more than 90%) and with a median interval of more than 30 months from PT diagnosis (3, 15, 16).

For the first time we analyzed immune infiltrates in both PT and BM in a series of CRC-derived BMs. We determined CD3 infiltration using artificial intelligence and the validated DGMate score, as well as CD8 infiltration, PD-L1 and PD-1 positive tumors (11, 12). CD3 and CD8 T-cell infiltration were higher in PTs as compared to BMs. To our knowledge, no other study has evaluated correlations between lymphocyte infiltration in BM and PT in CRC. Nevertheless, lymphocytes are typically absent from the healthy brain parenchyma (17). Most primary brain tumors contain few TILs, but some reports have suggested the presence of dense TIL infiltrates in BMs of different cancer types (9). In this series, most PTs were lung or breast cancers and correlation between PT and BM infiltrates was not analyzed. In a series of 46 matched samples of breast primary tumors and breast-derived BMs, BMs were positive for TILs in only 36% of cases compared to 82% of primary breast tumors (18). No correlation was established between CD3 infiltration in BMs and PTs in our series of CRC. To our knowledge, only one study has evaluated PD-L1 expression in BMs as compared to PTs in breast cancer and no difference was found between the two sites (18). In our series of CRC-derived BMs, PD-L1 overexpression was rare in both BMs and PTs. Moreover, PD-L1 overexpression in BMs was not associated with PD-L1 overexpression in PTs and vice versa. Angelova et al. analyzed both the clonal tumor cell evolution and immune landscape between PT and metastatic sites in two patients with metastatic CRC (19). They studied immune disparity from one metastatic site to another and showed that immunoediting is at work at the metastatic stage of CRC. The studied patients did not present BM but, in contrast to lung, breast cancers and melanoma, BMs represent a terminal evolution of CRCs. Immune response is expected to evolve drastically between the PT and the BM. Different rounds of chemotherapy may affect immune effectors, especially after a long disease history, which is the case in most CRC-derived BMs (20). These points could explain the absence of correlation of CD3 infiltration between BM and PT in our series of CRC-derived BMs. The lower CD3 T-cell infiltrates in BMs as compared to primary CRCs confirms the difficulties of T-cell recruitment in BMs.

In CRC, CD3 T-cell infiltrate in PTs has been associated with patient and tumor characteristics. Higher T and N stages have been associated with lower T-cell infiltration (11, 21). By contrast, in our study, we observed in PTs a trend of higher CD3 T-cell infiltration in higher T stages and no correlation with N stages. We also noted a non-significantly higher CD3 T-cell infiltration in rectal tumors compared to colon tumors. Our series was a subgroup of rare CRCs with BM whose T-cell infiltration had never been evaluated before. Moreover, CD3 staining covered different types of effector T-cells and infiltrates are different between tumor center and invasion front but in our series, only tumor center was studied (5, 11, 21, 22). Reichling et al. (11) evaluated CD3 T-cell infiltrates with the same method but only in stage III colon cancer, which is a different series compared to ours.

To our knowledge, no study has previously evaluated the correlation of CD3 infiltration with BM characteristics. We observed that CD3 T-cell infiltration in PT was higher when BMs were both supra and subtentorial. In addition, there was a trend of more CD3 T-cell infiltration in PT and BM when BMs were multiple. Caution and confirmation on a larger cohort are required to interpret these data given the low number of patients in each subgroup. Higher T-cell infiltration in BM could represent microenvironment modification due to cancer cell aggressiveness, which correlates with their ability to colonize both the infra and supra-tentorial brain.

DGMate score was first built and validated in stage III colon cancers (11). In this setting, DGMate score increased with T and N stages. In our series, DGMate score did not differ between PT and BM. Furthermore, we observed a strong correlation between the DGMate score in PT and BM, suggesting that this prognostic score had no major change during tumor progression. Our results indicate that although the immune environment is reshaped from PT to BM, other tumor characteristics taken into account by the DGMate score were stable despite time evolution and treatments. As in the PETACC08 study, there was a trend of higher DGMate score in PT with higher T stage. DGMate score was also higher in rectal tumors as compared to colon tumors. We also observed a trend of higher DGMate score in BM in patients with multiple BMs. These correlations suggest that DGMate score could be a surrogate marker of tumor aggressiveness in CRC-derived BM, as is already described in stage III CRC.

CD3 and/or CD8 T-cell infiltrates have been associated with CRC prognosis in many studies (5, 11, 21, 23). Nevertheless, in our series of CRC-derived BM, CD3 and CD8 infiltration in PT or BM did not correlate with OS. CD3 was questioned as a prognostic factor in locally advanced CRC and immune scores were developed to overcome the approximation linked to this single marker. More importantly, there is no strong evidence that CD3, CD8 or immune scores in PT of mCRC are predictive factors of OS. Several studies have shown discordance in immune cell infiltration between PT and metastases, but these studies focused mainly on liver or lung metastases (24–26). Moreover, some studies have suggested that the least immune-infiltrated metastasis predict OS in mCRC (10, 20, 26). These observations could explain why CD3 and CD8 infiltrates in PT or BM were not a prognostic factor in our series of mCRC with BM. As a positive CD3 staining covers different types of effector T-cells, some groups showed that a subgroup analysis of T-cells was required to establish a correlation of immune infiltration with prognosis at a metastatic stage, for instance in lung metastases (22). In contrast, in liver metastases, CD3 alone was correlated with OS (27). Furthermore, in a series of BMs derived from melanoma, lung, breast and renal cancers, the most frequently observed high immune infiltration involved CD3 positive cells and was associated with OS (9). In CRC, BM is a late event with poor prognosis. We analyzed only 37 BMs, which made correlations between T-cell infiltration in BM and OS difficult to establish. By contrast, PD-L1 positive PTs were associated with worse OS from CRC diagnosis. It is worth noting that only 5 PTs (6.8%) were PD-L1 positive. Our study showed comparable proportions of PD-L1 positive tumors when compared with other studies in the literature (28, 29). This association with OS should be interpreted with caution considering the small number of patients with PD-L1 positive tumors and potential tumor heterogeneity. High PD-L1 expression has been associated with longer OS in mCRC in some studies, but not all (30, 31). In addition, in lung cancers with BMs, PD-L1 expression has been associated with worse OS (30). Larger studies are needed to confirm the prognostic value of PD-L1 expression in cancer patients with BM.

DGMate score was associated with stage III colon cancer prognosis (11). In our cohort, DGMate score in PT did not correlate with OS. We hypothesized that the disease stage could account for this result since it was formed mainly of T3-T4 stage primary tumors (about 80%) and only stage III colon cancers by contrast to our series with only 34.1% of stage III CRC at diagnosis. At the BM site, DGMate score was not associated with OS. DGMate score is a tumor signature of 127 parameters whose interpretation might be intrinsic to each type of tissue and could differ from one type of tissue to another. Previously published prognostic factors in patients with BM from CRC were identified in our series, like ECOG PS and lung metastases (3).

Since CD3 T-cell infiltration and/or DGMate score were different according to PT site, BM site and BM number, we looked at a potential prognostic impact in these subgroups. High CD3 T-cell infiltration in BM was associated with lower OS in the subgroup of patients with supratentorial BMs. In addition, high DGMate score in BMs was associated with longer OS in two subgroups: patients with multiple BMs and patients with rectal tumor. Larger series are required to validate these associations. Supratentorial BMs were previously associated with better prognosis and multiple BMs with poor prognosis (3).

## Conclusion

CD3 and CD8 infiltration, PD-L1 expression and DGMate score at the brain metastatic site do not predict OS in patients with BMs from CRC. Our results suggest that immune response in CRC-derived BM differs from other CRC metastatic sites and further basic research focused on these lesions is required.

## Data Availability Statement

The raw data supporting the conclusions of this article will be made available by the authors, without undue reservation.

## Ethics Statement

The studies involving human participants were reviewed and approved by local ethics committee (DC-2008-565). Written informed consent for participation was not required for this study in accordance with the national legislation and the institutional requirements.

## Author Contributions

VR, LT, FG, and DT performed study concept and design. AD, SE, VD, PR, and JG performed development of methodology and carried out the experiments. AD, JG, and EF contributed to sample preparation. VR, FG, and DT contributed to the interpretation of the results. DT and VR took the lead in writing the first draft of the manuscript. All authors contributed to the article and approved the submitted version.

## Funding

This work was supported by a grant from the associations “Sport et Collection” and “Ligue Contre le Cancer, Comités départementaux de la Vienne, Charente et Charente-Maritime”.

## Conflict of Interest

The authors declare that the research was conducted in the absence of any commercial or financial relationship that could be construed as a potential conflict of interest.

## Publisher’s Note

All claims expressed in this article are solely those of the authors and do not necessarily represent those of their affiliated organizations, or those of the publisher, the editors and the reviewers. Any product that may be evaluated in this article, or claim that may be made by its manufacturer, is not guaranteed or endorsed by the publisher.
